# Critical Frequency of Self-Heating in a Superelastic Ni-Ti Belleville Spring: Experimental Characterization and Numerical Simulation

**DOI:** 10.3390/s21217140

**Published:** 2021-10-27

**Authors:** Emmanuel Ferreira de Souza, Paulo César Sales da Silva, Estephanie Nobre Dantas Grassi, Carlos José de Araújo, Antonio Gilson Barbosa de Lima

**Affiliations:** Multidisciplinary Laboratory of Active Materials and Structures (LaMMEA), Department of Mechanical Engineering, Federal University of Campina Grande, Campina Grande 58429-140, Brazil; emmanuelgafa@gmail.com (E.F.d.S.); end.grassi@hotmail.com (E.N.D.G.); carlos.jose@professor.ufcg.edu.br (C.J.d.A.); antonio.gilson@ufcg.edu.br (A.G.B.d.L.)

**Keywords:** shape memory alloys, superelasticity, Ni-Ti Belleville spring, self-heating frequency, dynamic loading

## Abstract

The mechanical loading frequency affects the functional properties of shape memory alloys (SMA). Thus, it is crucial to study its effect for the successful use of these materials in dynamic applications. Based on the superelastic cyclic behavior, this work presents an experimental methodology for the determination of the critical frequency of the self-heating of a NiTi Belleville conical spring. For this, cyclic compressive tests were carried out using a universal testing machine with loading frequencies ranging from 0.5 Hz to 10 Hz. The temperature variation during the cyclic tests was monitored using a micro thermocouple glued to the NiTi Belleville spring. Numerical simulations of the spring under quasi-static loadings were performed to assist the analysis. From the experimental methodology applied to the Belleville spring, a self-heating frequency of 1.7 Hz was identified. The self-heating is caused by the latent heat accumulation generated by successive cycles of stress-induced phase transformation in the material. At 2.0 Hz, an increase of 1.2 °C in the average temperature of the SMA device was verified between 1st and 128th superelastic cycles. At 10 Hz, the average temperature increase reached 7.9 °C and caused a 10% increase in the stiffness and 25% decrease in the viscous damping factor. Finally, predicted results of the force as a function of the loading frequency were obtained.

## 1. Introduction

Shape memory alloys (SMA) are smart metallic materials with the ability to reverse considerable mechanical deformation (in the order of 10% in tension for NiTi alloys), even under substantial mechanical loading [1]. The shape recovery can occur through two distinct phenomena: shape memory effect (SME), taking place when heating is applied after a pseudoplastic deformation; and superelasticity (SE), an isothermal loading/unloading cycle leading to a pseudoelastic deformation. Both SME and SE are associated with energy dissipation, observable through a mechanical hysteresis between loading and unloading curves. These phenomena occur due to a reversible solid-state phase transformation between the crystallographic phases austenite (A) and martensite (M). As in all phase changes, the phase transition in SMA is associated with the release of latent heat, being exothermic during the forward transformation (A → M), and endothermic during the reverse transformation (M → A).

The mechanical behavior of SMA is intimately related to temperature. The stress to mechanically trigger the phase transformation in SMA increases linearly with temperature, following a Clausius–Clapeyron law [2,3,4]. This means that SMA becomes stiffer as the temperature increases. Besides the surrounding medium temperature, the latent heat generated during phase transformation can interfere with the mechanical behavior. At low loading frequencies, the latent heat has a negligible effect on the SMA behavior since it is easily dissipated to the medium. During cyclic loadings, however, the generated latent heat might accumulate if the loading frequency is sufficiently high, which in turn causes the material to stiffen as loading progresses due to the Clausius–Clapeyron relation. This thermomechanical coupling is the cause of the strain rate dependence observed in SMA [5,6,7].

High loading frequencies are characteristic of dynamic applications, which might require vibration damper systems. For these situations, superelastic SMA has great potential due to the material’s intrinsic energy dissipation capacity. Some examples of the dynamic applications of SMA include re-centering devices in civil engineering [8,9,10,11] and vibration dampers applied in varied mechanical systems, such as rotary machines [12,13,14]. Nevertheless, in these dynamic applications, functional thermomechanical properties, such as energy dissipation capacity and equivalent viscous damping factor can be considerably hindered by the effects of self-heating due to the accumulation of latent heat during cyclic phase transformation, leading to overall inefficiency. Therefore, the identification of parameters that induce self-heating, especially loading frequency, is crucial for the functionality of SMA devices working under cyclic loadings.

One such device is a conical spring, known as Belleville spring or Belleville washer, engineered to work as compact spring elements, able to bear high compression forces with little deformation compared to other spring geometries. Belleville springs or washers are also widely used components in machinery, automotive, civil structures and in many other fields as vibration damping elements and fastening devices [15,16,17,18]. When manufactured with SMA, the combined geometry and functional material properties result in a mechanical component with superelasticity and improved damping capacity. Several investigations have been devoted to the study of the thermomechanical behavior of SMA-based Belleville washers [19,20,21], especially in the development and optimization of seismic resisting applications [10,11,22,23,24]. However, these studies do not consider the effects of the thermomechanical coupling of the SMA material, which certainly plays a role in the dynamic behavior of SMA Belleville devices.

In this context, this work proposes an experimental methodology for the characterization of thermomechanical coupling during the dynamic loading of NiTi SMA Belleville springs. The methodology focuses on the analysis of the influence of the load frequency in thermomechanical behavior and determines the critical frequency of self-heating as a consequence of the accumulation of latent heat. A numerical simulation of the isothermal behavior complements the analysis, allowing for the estimation of the force increase to reach a determined imposed strain, caused by the self-heating phenomenon.

## 2. Methodology

### 2.1. Experimental Characterization

#### 2.1.1. Material and Spring Manufacturing

To perform the experiments, a NiTi SMA Belleville spring was manufactured from a commercial NiTi sheet with a thickness of 0.8 mm and nominal composition of 55.57% Ni-Ti (%wt), supplied by Sunrise Titanium Technology Company (Shaanxi, China). The NiTi sheet was cut by wire electro-discharge machining (EDM) in a flat circular disk with 25.4 mm and 7.0 mm of outer and inner diameters, respectively. An annealing treatment at 500 °C for 60 min was performed on the NiTi disk, as recommended by the NiTi sheet manufacturer for stress relief.

**Figure 1:**
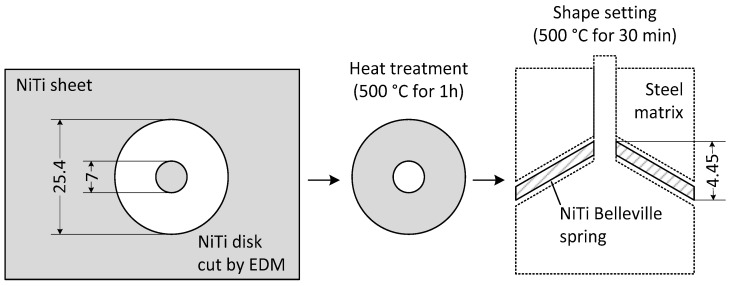
Fabrication process of the NiTi SMA Belleville spring. Dimensions in mm.

To achieve the conical shape of the Belleville spring, a shape setting was performed at 500 °C for 30 min. The shape was impressed on the NiTi disk by a steel matrix. The resulting outer/inner diameter ratio was approximately 3.6 and the free height/thickness ratio was 4.6. A single SMA Belleville spring was manufactured and Figure 1 shows schematically the manufacturing process and the spring dimensions.

#### 2.1.2. Thermal Characterization of the NiTi Belleville Spring

The transformation temperatures of the NiTi SMA were determined by Differential Scanning Calorimetry (DSC) (model Q20 from TA Instruments, New Castle, DE, USA). The used temperature range was −70 °C to 100 °C with a cooling/heating rate of 10 °C/min under a nitrogen atmosphere (ASTM F2004-17). Since the DSC technique is based on heat exchanges, it requires a small sample to guarantee a uniform heat distribution during tests. Therefore, the DSC analysis was performed on a small sample taken from the Belleville spring material after its manufacturing process. The starting and final phase transformation temperatures, both during forward and reverse martensitic transformation, were obtained by the tangent intersection method applied to the DSC peaks.

#### 2.1.3. Mechanical Characterization of the NiTi Belleville Spring

**Figure 2:**
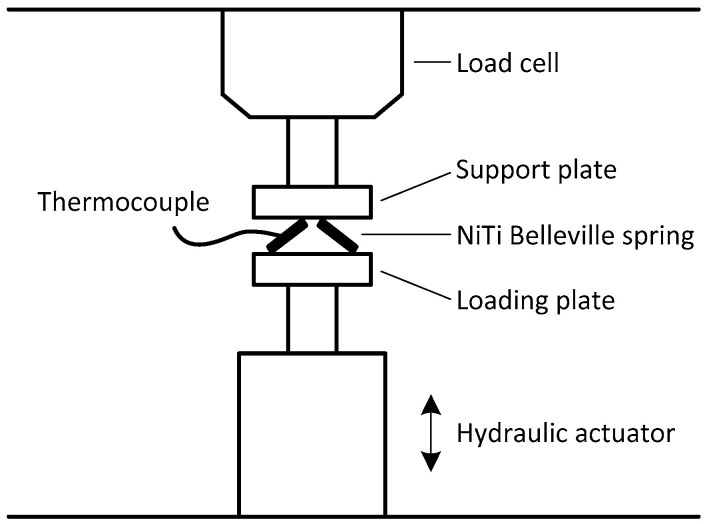
Experimental setup for cyclical compressive tests using MTS machine.

Prior to the dynamic tests, a quasi-static pre-characterization was performed on the SMA Belleville spring at different temperatures. The results were used to determine the suitable pre-deflection level for the dynamic tests (small enough to avoid the support plate (see Figure 2) losing contact with the Belleville spring during tests, while being large enough to allow a good displacement range and therefore maximize SMA phase transformation and consequently the self-heating effects). Under a sinusoidal displacement wave at a low frequency of 0.1 Hz, the spring was submitted to deflections of 75% of its free height at 35 °C, 45 °C, and 55 °C. The temperature was controlled in the clamping zone of the testing machine by an environmental chamber with a resistive heating system.

To investigate the thermomechanical coupled behavior of the NiTi Belleville spring, cyclic compressive tests were carried out using a servo-hydraulic universal testing machine (model 793 from MTS) equipped with a 100 kN loading cell. Prior to the characterization tests, the Belleville spring was submitted to a pre-cycling with 50 compressive cycles at 0.1 Hz to minimize the evolution of the superelastic behavior. The superelasticity is known to rapidly evolve during the first cycles and achieve a more stabilized behavior after a certain number of cycles is achieved [10,25,26], which naturally depends on the sample geometry and strain amplitude. After the pre-cycling, the NiTi Belleville spring was submitted to 128 compressive sinusoidal cycles at 11 loading frequencies between 0.5 Hz and 10 Hz to evaluate the influence of loading frequency on the superelastic cyclic behavior. Prior to each cyclic test, the temperature of the sample was stabilized at room temperature to avoid the influence of heat accumulation from the previous test. Both pre-cycling and dynamic characterization were performed within the same deflection range, determined in the loading at 0.1 Hz.

The temperature variation during the cyclic tests was monitored using a K-type micro thermocouple (100 μm in diameter) glued to the outer surface of the NiTi Belleville spring. Temperature evolution was recorded by a data acquisition system set with an acquisition rate of 100 Hz (model Quantum X from HBM). Figure 2 shows a schematic drawing of the experimental setup used in mechanical tests. A thin layer of lubricating oil to reduce friction was used between the device and the compression plates.

### 2.2. Numerical Simulation

Finite element analysis (FEA) was performed using Auricchio’s superelastic material model available on the commercial ANSYS Mechanical software v17 package. This classical superelasticity model has been available on ANSYS Mechanical since the 14.5 version (2012) and its constitutive laws are described in detail in [27]. The model considers the total recovery of pseudoelastic strain under isothermal conditions and large-deformation. The material parameters of the model are the austenite elastic modulus (E), Poisson’s ratio (ν), starting and final stresses for the forward and reverse transformations ($\sigma_{s}^{\mathrm{AM}}$, $\sigma_{f}^{\mathrm{AM}}$, $\sigma_{s}^{\mathrm{MA}}$ and $\sigma_{f}^{\mathrm{MA}}$), maximum residual strain ($\epsilon_{L}$), and a parameter that measures the tension-compression asymmetry of the material (α). These parameters were determined indirectly by manually adjusting the numeric results to the experimental superelastic loops obtained at 35 °C, 45 °C, and 55 °C.

**Figure 3:**
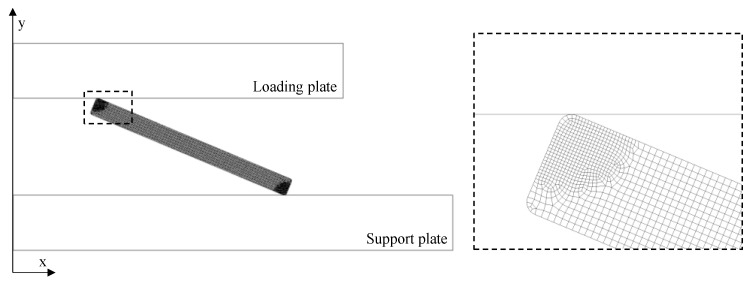
2D axisymmetric model of the Belleville spring discretized, showing mesh detail.

A 2-D axisymmetric model of the NiTi Belleville spring was discretized with an unstructured mesh with a high-order 2-D plane 8-nodes element that exhibits quadratic displacement behavior and supports large strains. Two-dimensional 3-node surface-to-surface contact is used to represent contact and sliding between the 2-D target surfaces. The CAD model of the conical spring was discretized with an average size of 0.05 mm and refined contact regions with an average size of 0.025 mm, resulting in a total of 11,812 nodes, as shown in Figure 3.

Boundary conditions restrict all degrees of freedom of the nodes on the support plate while applying a prescribed displacement to the loading plate. Because lubricating oil is used in the experiments, a low friction contact is defined with a coefficient of 0.11 [28] between the upper and lower rounded corners (radius of 0.1 mm) of the NiTi Belleville spring and the loading and support plates (structural steel), respectively; with symmetric behavior, augmented Lagrange formulation, and an on-Gauss point detection method. Thermal effects are disregarded in this model.

## 3. Results and Discussion

### 3.1. Experimental Analysis

#### 3.1.1. Transformation Temperatures

**Figure 4:**
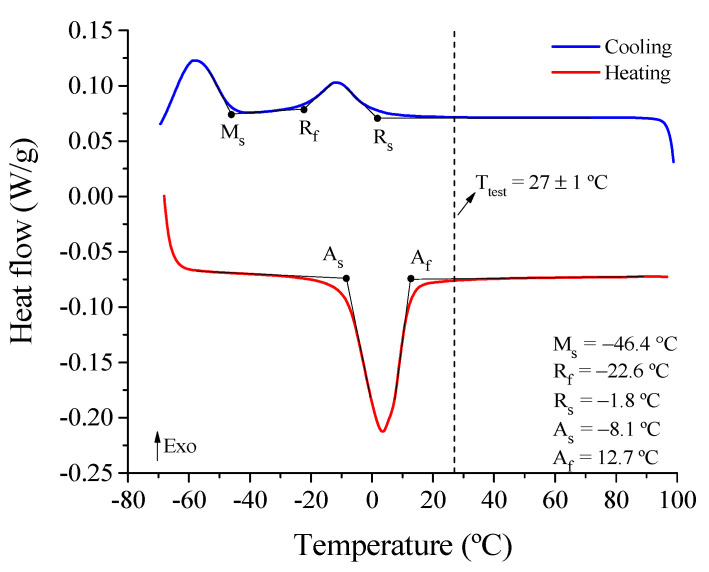
DSC result of the NiTi Belleville spring.

As observed in Figure 4, the NiTi SMA presents a stable austenite phase from about 12.7 °C. Therefore, under test conditions at room temperature (27 ± 1 °C), the NiTi Belleville spring should predominantly have an austenite phase and therefore exhibit superelastic behavior. The DSC results also show that the reverse phase transformation takes place in two steps, austenite B2 to R-phase and R-phase to monoclinic martensite B19′. The M_f_ temperature is not determined due to an incomplete phase transformation between R-phase and monoclinic martensite B19′.

#### 3.1.2. Superelastic Response at Low Loading Frequency

**Figure 5:**
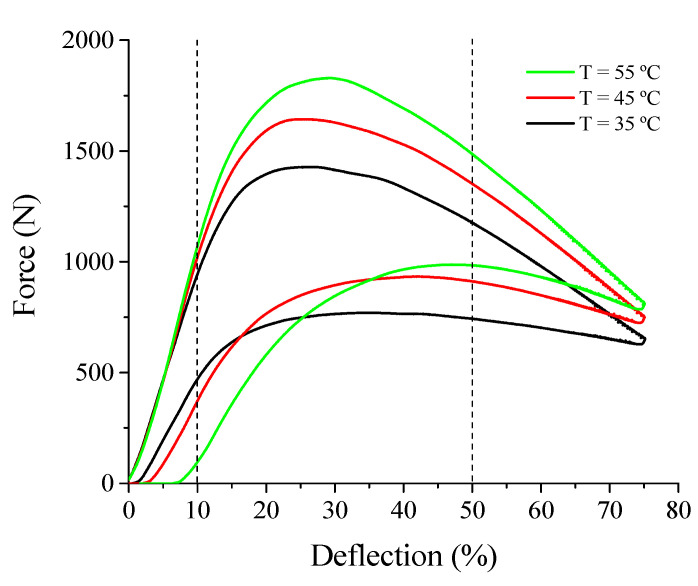
Isothermal superelastic response at low frequency (0.1 Hz) of the NiTi Belleville spring observed in force vs. deflection curves. The vertical lines indicate the deflection range selected for the cyclic compression tests.

Figure 5 shows force vs. deflection superelastic loops at 35 °C, 45 °C, and 55 °C performed at 0.1 Hz. With increasing temperature, the superelastic loop moves up, increasing peak force from 1428 N (35 °C) to 1829 N (55 °C). This behavior follows the Clausius–Clapeyron law, as explained by Savi et al. [29]. The evolution of the accumulated strain is also observed with increasing temperature.

For applications of SE SMA devices in the dynamic regime, pre-static or low loading frequency characterization is useful for the identification of the pre-load or pre-displacement suitable for the application. Once the maximum oscillation amplitude is defined from the design criteria, the designer can select the preload or pre-displacement level to obtain an optimized response from the SMA device regarding its energy dissipation capacity (mechanical hysteresis). From the results shown in Figure 5, deflection ranges from 10% to 50% were selected as the oscillation amplitude. The 10% preload was also useful in avoiding the need to correct the distance between the compression plates to account for any strain accumulation of the SMA spring during cyclic tests.

For a linear-elastic material, the higher the free height/thickness ratio of a Belleville spring, the more pronounced was the drop in force after the peak [30]. However, the use of a superelastic material provided the shape recovering during unloading, generating a force vs. deflection curve named by [24] as “duck-head-shaped”. This behavior depends not only on geometric parameters, such as free height/thickness ratio, outer/inner diameter ratio and cone angle, but also on the level of the applied loading and still requires more investigation.

#### 3.1.3. Mechanical Pre-Cycling

**Figure 6:**
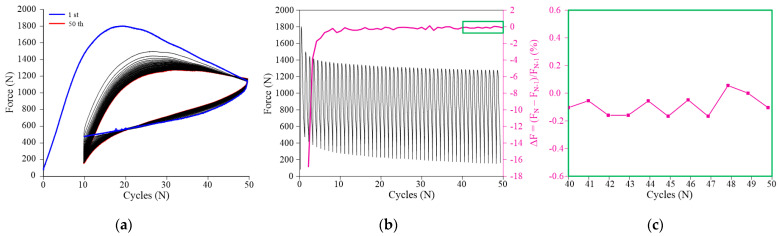
Mechanical behavior during the pre-cycling (at 0.1 Hz) of the superelastic behavior. (**a**) Force vs. deflection response. (**b**) Force vs. cycles response (left axis) and percentage force variation in relation to the N-1 cycle (right axis). (**c**) Detail of the percentage force variation.

The mechanical pre-cycling of SMA is an essential step for this study. It minimizes the dependence of the NiTi Belleville spring mechanical behavior, and consequently of its functional properties, from the number of cycles. The pre-cycling process is shown in Figure 6a. The superelastic loop evolves and tends to stabilize after a certain number of cycles due to the saturation of the forward and reverse phase transformations, presenting a gradual decrease in force. Figure 6b (left axis) shows the force variation with cycling. The variation of the force peaks in relation to the previous cycle (F_N_ − F_N−1_/F_N−1_) is also shown in this figure (right axis). As typically observed in SMA cycling [10,25,26], the evolution of the superelastic behavior is not linear and the bulk of the force decrease occurred within the first cycles. Indeed, the force variation curve in Figure 6b shows a more accentuated decrease in the first cycles (303 N, ~18%), which becomes smaller as cycling progresses (1.1 N on average between the 40th and 50th cycles, or ~0.2%, as shown in detail in Figure 6c). Similar mechanical behavior was observed by [10,31]. The force decrease is due to the redistribution of internal stresses during stress-induced phase transformation, mainly caused by the accumulation of dislocation slip [32,33,34]. Diffraction patterns of NiTi wires under tensile loading show that this gradual plastic deformation generates a gradual accumulation of defects and residual martensite, and an increase of the volume fraction of non-transforming austenite, consequently decreasing the amount of stress-induced martensite with each new cycle [34].

#### 3.1.4. Superelastic Response and Thermomechanical Coupling in the Dynamic Regime

**Figure 7:**
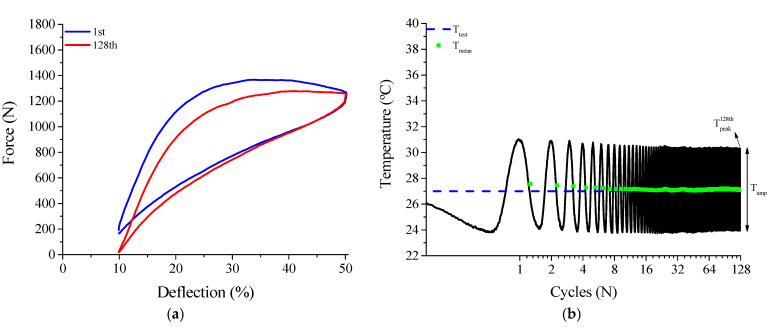
Thermomechanical behavior at 0.5 Hz. (**a**) Superelastic dynamic response in the 1st and 128th cycles. (**b**) Temperature response during cycling.

After the pre-cycling of the superelastic behavior of the NiTi Belleville spring, the influence of the thermomechanical coupling on the superelastic cyclic behavior was investigated. Figure 7a,b show force vs. deflection and temperature vs. cycle behavior, respectively, for the frequency of 0.5 Hz. A force decrease is observed between the 1st and 128th cycles, with peak force values of 1367 N and 1277 N, respectively, corresponding to a reduction of approximately 6.5%.

At this low frequency, the temperature signal mostly oscillated around the test temperature (*T_test_*), measured as the initial value of the temperature signal (~26 °C). The mean temperature (*T_mean_*) decreases slightly at the end of cycling at 0.5 Hz. Peak temperature ($T_{peak}^{128\mathrm{th}}$) and amplitude temperature (*T_amp_*) by the end of the 128th compressive cycle was 30.3 °C and 6.4 °C, respectively.

The force reduction observed at 0.5 Hz might still be associated with the continuous evolution of mechanical cycling, even though the pre-cycling promoted the better part of this microstructural evolution. On the other hand, it is worth noting that the thermomechanical coupling of the SMA material is always active during phase transformation. As the temperature reached at the end of the superelastic cycles was lower than room temperature, the material started each new cycle in a softer state compared to the first cycle due to the Clausius–Clapeyron relation. Therefore, thermomechanical coupling effects are very likely playing a role in the force decrease between the 1st and 128th cycles at lower loading frequencies.

**Figure 8:**
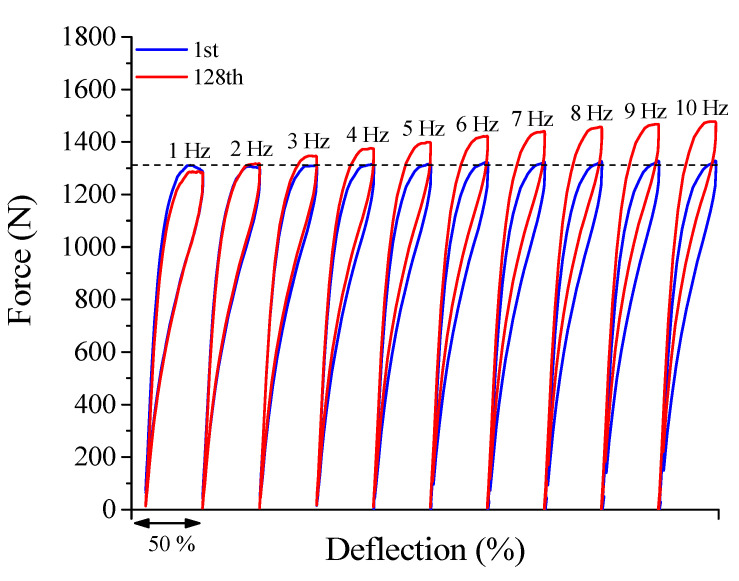
Dynamic response of the superelastic behavior for loading frequencies from 1 Hz to 10 Hz. The horizontal line is used to compare the force levels at the beginning of each cycle.

Figure 8 shows the superelastic loops of the 1st and 128th cycles of the NiTi Belleville spring for frequencies from 1 Hz to 10 Hz. In the studied frequency range, 1 Hz is the only frequency where the peak force decreases, from 1311 N to 1287 N, a reduction of approximately 1.8%. The horizontal line is used to show that the first cycles (in blue) do not show any sign of decrease due to cycling effects. Moreover, the maximum force of the first cycle at 2 Hz is higher than the last cycle at 1 Hz. This points to a thermomechanical coupling effect similar to that observed at 0.5 Hz, causing the force decrease between the 1st and last cycles at the lower frequencies.

From 2 Hz the peak force value for the 128th cycle increases in relation to the 1st cycle. This behavior is intimately linked to the accumulation of latent heat during successive phase transformation cycles under high loading frequencies, leading to an increased internal temperature of the NiTi alloy and a consequent increase in stiffness due to the Clausius–Clapeyron relation.

**Figure 9:**
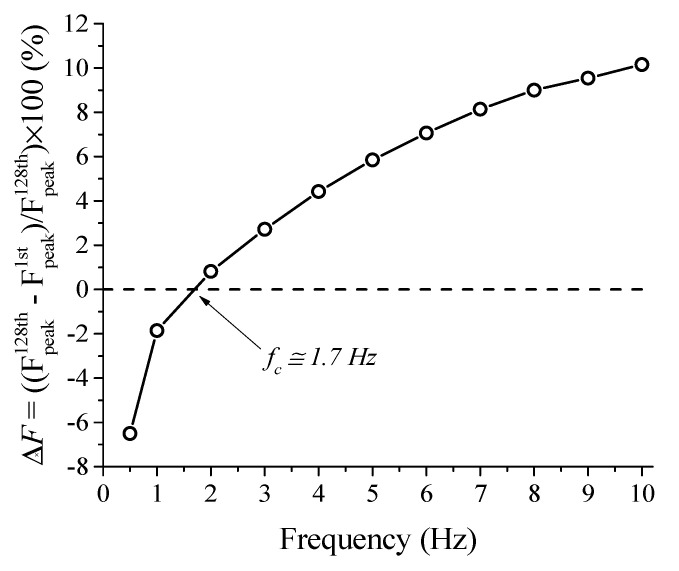
Determination of the critical frequency of self-heating for the SMA Belleville spring.

From the force vs. deflection superelastic response, the percentage force difference (Δ*F*) between the 1st and 128th cycles is measured and shown in Figure 9. For the frequencies of 0.5 Hz and 1 Hz, the Δ*F* value is negative, i.e., the peak force value for the 128th cycle is lower than the peak force value for the 1st cycle. For frequencies from 2 Hz, Δ*F* the value is positive, increasing monotonically with increasing rates. At 10 Hz, the Δ*F* value reaches approximately 10%, i.e., the peak force of the last cycle is 10% higher than the force of the 1st cycle.

The dashed line delimits Δ*F* values as positive or negative. In this work, we propose that the intersection between Δ*F* = 0 line and the Δ*F* curve for various cycling frequencies identifies the self-heating frequency (*f_c_*). If the SMA material or device is cyclically loaded at frequencies above *f_c_*, the SMA will accumulate latent heat and consequently become stiffer. The *f_c_* value for the NiTi Belleville spring analyzed in this work is 1.7 Hz. Note that this value is dependent on specific convective and conduction heat transfer conditions and amplitude levels. Changes in the geometrical parameters of the Belleville spring will change the stress-induced martensite fraction and consequently will influence the heat exchange by conduction between the spring and the compression plates and by convection, between the spring and the environment. If the heat exchanges are intensified, for example by increasing the convection coefficient, the self-heating frequency would increase, since the material would be capable of dissipating the generated heat.

**Figure 10:**
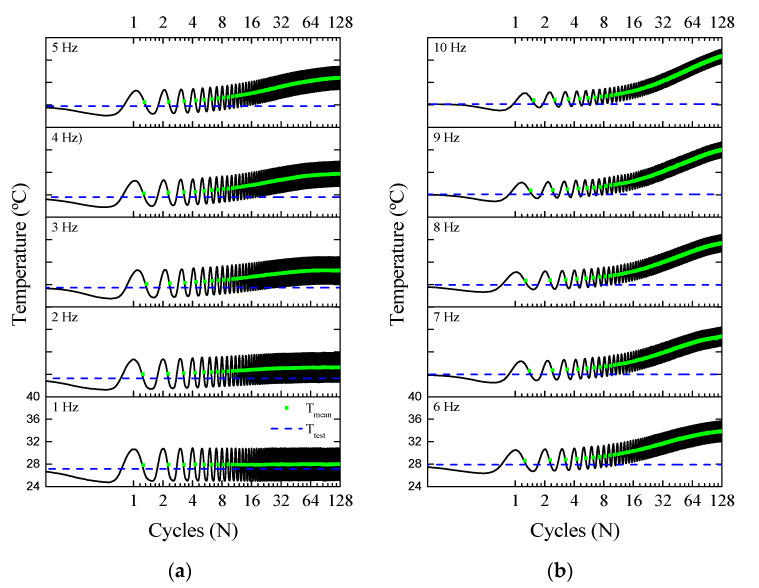
Temperature response over cycles. (**a**) from 1 Hz to 5 Hz. (**b**) from 6 Hz to 10 Hz.

Figure 10 demonstrates the self-heating phenomenon in the NiTi Belleville spring by showing the temperature signal throughout cycling. The force behavior observed in Figure 8 is related to this self-heating, where SMA accumulates the latent heat generated due to the forward phase transformation (A → M) that is not completely dissipated during the reverse phase transformation (M → A). Thus, it is possible to state that there is a critical frequency between 1 Hz and 2 Hz where the Belleville spring will start to self-heat, which will increase the overall stiffness of the dynamic system and diverge from the designed working parameters.

From the temperature evolutions in Figure 10, two main observations can be made: first is the variation of *T_mean_*, which increases from 2 Hz; and the decrease in *T_amp_* with increasing frequency. For instance, at the 128th cycle, *T_mean_* reaches 29.3 °C at 2 Hz and 36.7 °C at 10 Hz. The number of cycles was not enough to stabilize *T_mean_*, and it is assumed that *T_mean_* would further increase before stabilizing, reaching thermal equilibrium.

**Figure 11:**
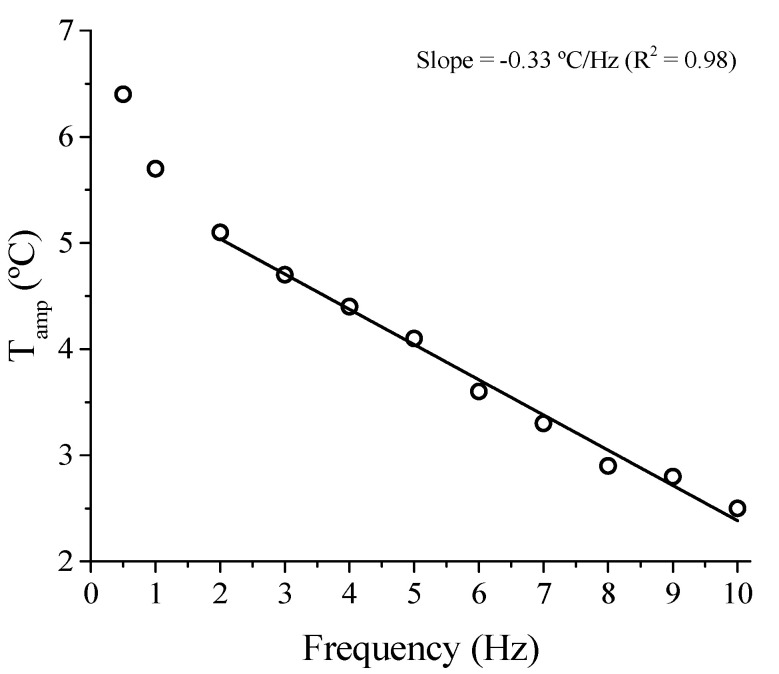
Temperature amplitude (*T_amp_*) of the NiTi Belleville spring as a function of loading frequency.

The two aforementioned observations are looked at in more detail in Figure 11. It is observed that *T_amp_* (for the 128th) decreases almost linearly with increasing frequency, decreasing by half between 2 Hz and 10 Hz (5.1 °C and 2.5 °C, respectively). We observed a linear decreasing relationship between the *T_amp_* and the frequency from 2 Hz, with an angular coefficient of −0.331 °C/Hz, being able to estimate with good precision (R^2^ = 0.98) the *T_amp_* from a certain frequency. Although this coefficient is valid for the entire system (Belleville spring, compression plates, atmosphere), its value provides an order of magnitude for the relationship between temperature and loading frequency. Furthermore, the fact that the Belleville spring is not insulated is much closer to the environment found in industrial applications.

#### 3.1.5. Functional Properties

From the superelastic force vs. deflection response of the NiTi Belleville spring, some functional properties, such as secant stiffness (*k_s_*, in kN/m), dissipated energy per cycle (*E_D_*, in MJ/m^3^), and equivalent viscous damping factor (*ξ*, in %) can be obtained. These functional properties are useful for the design of devices manufactured from SMA, especially when the envisioned application works under a dynamic regime.

Secant stiffness *k_s_* is calculated by the ratio between the difference of peak and valley forces (∆*F*) and the displacement (∆*δ*) related to deflection between 10% and 50%, as expressed by Equation (1).

(1) $$k_{s}=\frac{\Delta F}{\Delta \delta }$$

with ∆*δ* being 1.48 mm, which is equivalent to 40% of the free height of the NiTi Belleville spring.

**Figure 12:**
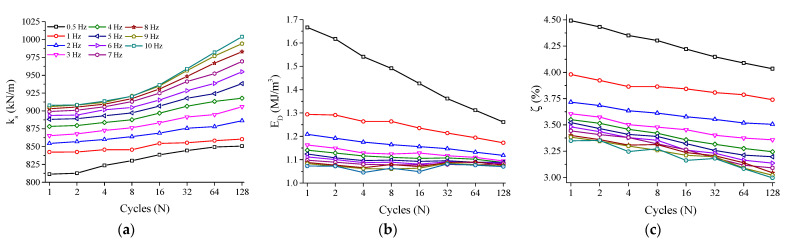
Functional properties over cycles as a function of the load frequencies analyzed. (**a**) Secant stiffness. (**b**) Energy dissipated per cycle. (**c**) Equivalent viscous damping factor.

Figure 12a shows the behavior of *k_s_* vs. frequency. The cycles are plotted at each 2*^n^* cycle (*n* = 0, 1, 2, 4, …, *n*) to facilitate visualization. *k_s_* increases with cycling for all the analyzed frequencies. The rate of this increase, however, changes at different frequencies. Mainly for frequencies of 4 Hz and above, the rate of increase becomes stronger at the 8th cycle. This behavior is a consequence of the increase in the internal temperature after the 8th cycle (see Figure 10), which stiffens the NiTi Belleville spring. The percentage increase of *k_s_* during the performed cycling went from 4.8% at 0.5 Hz to 10% at 10 Hz.

Analogously to Figure 12a, Figure 12b shows the behavior of *E_D_* with cycling for the analyzed frequencies, which is calculated using Equation (2) through the integration of the force vs. displacement superelastic loop divided by the volume of the Belleville spring (obtained from the CAD model). Overall, *E_D_* decreases linearly with both cycling and with the increase of frequency.

(2) $$E_{D}=\frac{\oint Fd\delta }{Volume}$$

At 0.5 Hz, the decrease is much more pronounced, most likely due to the evolution of mechanical cycling, which could still be taking place at this first tested frequency. While the observed reduction of *E_D_* reached 24% for the cycling at 0.5 Hz (from 1.66 MJ/m^3^ to 1.26 MJ/m^3^); it was only 0.2% at 10 Hz. As for the variation with the frequency, the *E_D_* variation between 2 Hz and 10 Hz at the last cycle reached 4.5% (1.12 MJ/m^3^ and 1.07 MJ/m^3^, respectively). The energy dissipation capacity of SMA is mostly originated by the friction between transforming regions and the movement of defects in the crystal lattice [4]. In quasi-static loadings, a hypothesis for the decrease in energy dissipation capacity with increasing temperature is that higher temperatures lead to higher transformation stresses (due to the Clausius–Clapeyron relation), and consequently more favorably oriented martensite variants are formed in detriment of the less favorably oriented ones (due to the stronger stress field) [3]. If this is the case, less internal lattice movement occurs, causing less energy loss.

The viscous damping factor ζ, which is another parameter used in the mechanical design of devices manufactured with SMA, is calculated as a ratio of the dissipated energy per cycle to the dissipated energy of an equivalent linear element (*E_so_*), as expressed by Equation (3) [24].

(3) $$\zeta =\frac{E_{D}}{4\pi E_{SO}}\times 100$$

Figure 12c shows the variation of ζ with cycling and frequency, which is directly related to *E_D_* and, therefore, follows the same behavior. The highest values are observed for the frequency of 0.5 Hz, which are 4.49% and 4.03%, for the 1st and 128th cycles, respectively, while the lowest values are observed for the frequency of 10 Hz, which are 3.34% and 2.99% for the 1st and 128th cycles, respectively. According to [24], values around 5–10% are typical in structural engineering.

### 3.2. Numerical Analysis

The mechanical behavior of a device is a sum of material and geometric features. If, due to geometry, the deformation of the SMA is not uniform throughout the mechanical part, neither is the phase transformation that originates superelastic behavior. To assist with the analysis of the NiTi Belleville spring mechanical behavior, a numerical simulation is used to access the martensite fraction distribution when it is mechanically loaded.

**Figure 13:**
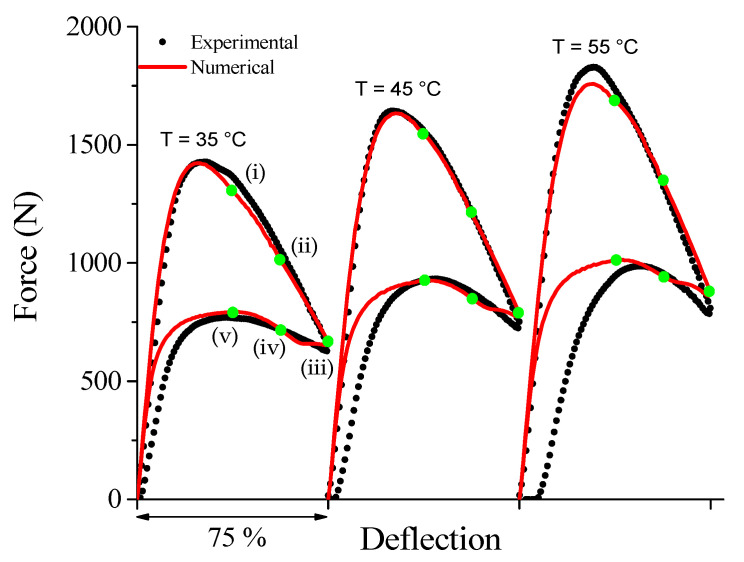
FEA and experimental superelastic response at low loading frequency under controlled temperature.

The ideal material model for the numerical analysis of the NiTi Belleville spring under adiabatic conditions would have to consider the influence of the loading frequency and the accumulation of latent heat with cycling. However, although the material model restricts numerical analysis to isothermal conditions, we observe a good agreement between the FEA and experimental superelastic responses, as shown in Figure 13. The mechanical properties used to feed the FEA model are shown in Table 1.

FEA captures the superelastic response of force vs. deflection for the three analyzed temperatures. Although the model does not capture the accumulated strain observed in the unloading portion of the curve as temperature increases, the evolution of the loading curvature (duck-head-shape) with increasing temperature is well reproduced by the model.

**Figure 14:**
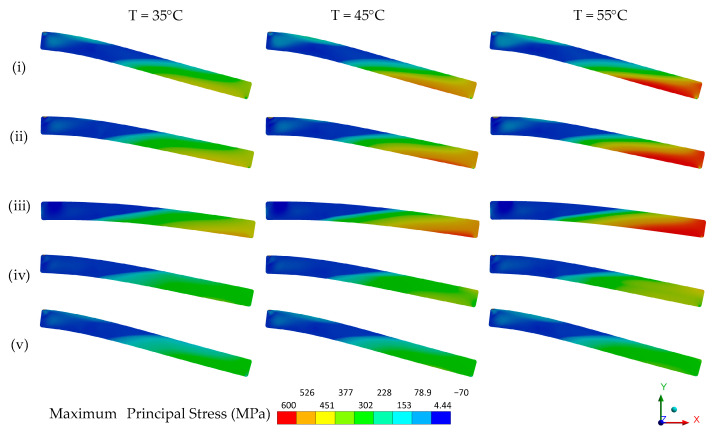
Contour plot of the hoop stress in NiTi Belleville spring.

**Figure 15:**
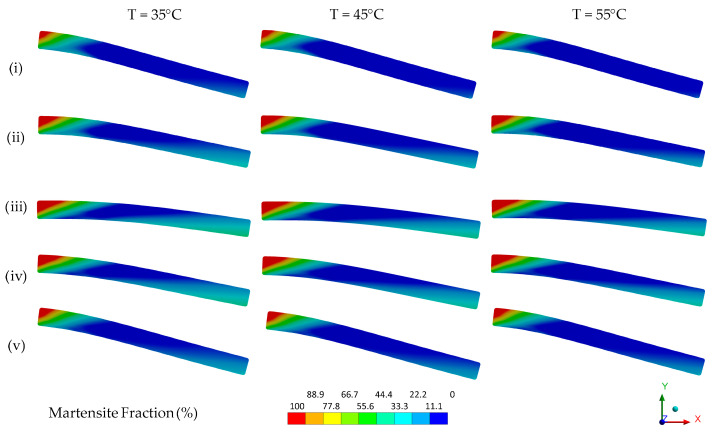
Contour plot of the stress-induced martensite fraction (%) in NiTi Belleville.

Figure 14 and Figure 15 show respectively the contour plots of the hoop stress and the stress-induced martensite fraction in the NiTi Belleville spring at four instants, highlighted in green in the superelastic response shown in Figure 13. In general, the regions near the support plate (bottom) reach the highest hoop stress values, while the regions close to the loading plate (top) reach the lowest hoop stress values. Furthermore, we observe that the hoop stress gradients are different during loading and unloading, although the selected instants correspond to similar deflections, indicating a non-linear stress distribution.

The regions near the loading plate (top) achieved 100% of martensite fraction for all instants, while the regions near the support plate (bottom) reach only 35% at instant (iii). In the central region of the Belleville spring, the martensite fraction is zero at all instants, and, therefore, there is no phase transformation in this zone for the imposed deflection. At a fixed instant, we observe that as the temperature increases and the material becomes stiffer due to the Clausius–Clapeyron law, the observable phase transformation zones decrease in size since more mechanical energy is required to trigger the phase transformation. Furthermore, the heterogeneous distribution of the martensite fraction is an indication that the temperature of the NiTi Belleville spring is not uniform throughout its cross-section, even under quasi-static conditions. According to Figure 15, there is likely a strong heat flow from the top to the central region of the spring in the conditions of the performed test (heat balance between SMA, compression plates, and environment at the beginning of test and natural convection).

**Figure 16:**
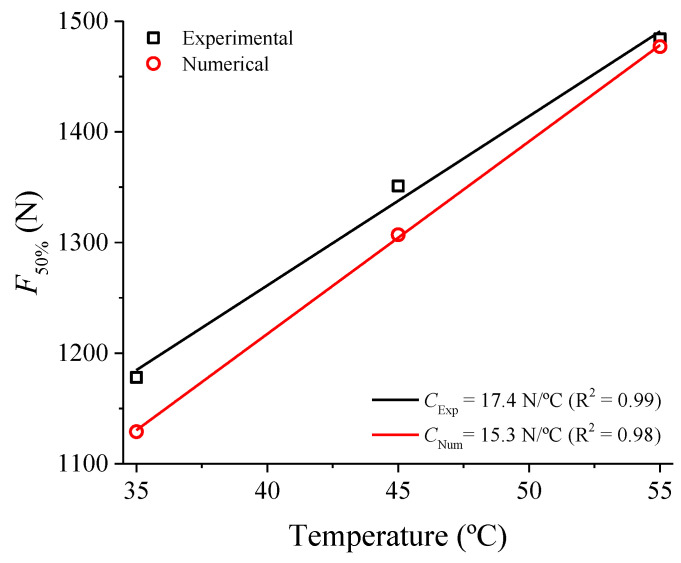
Experimental vs. numerical linear increase of *F*_50%_ as a function of temperature.

From Figure 13 we calculate the Clausius-Clayperon coefficient of force increase as a function of temperature (*C*) using the force corresponding to a deflection of 50% in Equation (4), the theoretical value of 15.3 N/°C is obtained using numerical simulation data, as pointed out in Figure 16.

(4) $$C=\frac{\Delta F_{50\%}}{\Delta T}$$

**Figure 17:**
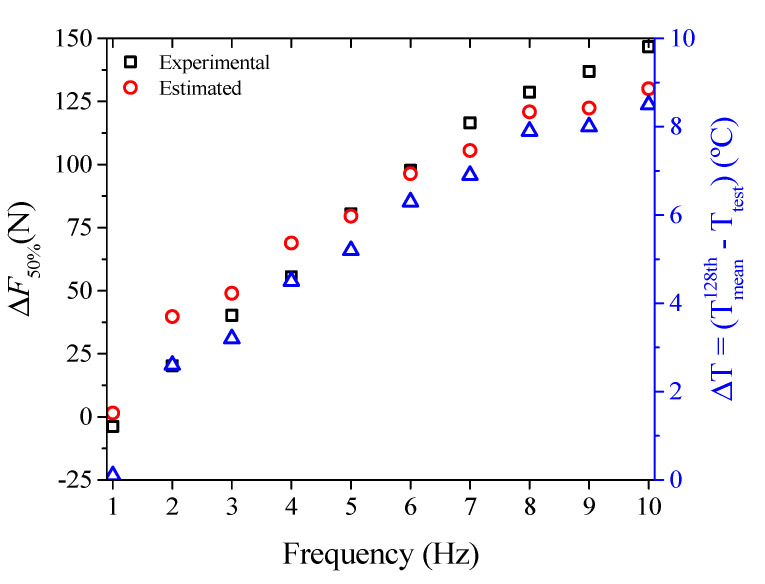
Estimated vs. experimental force increase Δ*F*_50%_ as a function of loading frequency.

With the value of the theoretical constant *C* and the measured temperature rise Δ*T* = *T* − *T_mean_* from Figure 10, it is possible to make a simplification to estimate the force increase at 50% deflection, Δ*F*_50%_, using Equation (4) (Δ*F*_50%_ = *C*(*T* − *T_mean_*)). This estimated increase in force can be compared with the experimental observation in Figure 9 for the frequencies at which self-heating occurs (from 2 Hz). Figure 17 shows the results of this comparison.

Figure 17 shows that the higher the loading frequency from the self-heating frequency (~1.7 Hz), the more the theoretical estimate for Δ*F*_50*%*_ approaches the experimental value, up to 6 Hz. Between 7 Hz and 10 Hz, the estimated theoretical value is always lower than the experimental one, but this is because the mean temperature (*T_mean_*, Figure 10) is not stabilized, but still increasing. In other words, with the *T_mean_* value already stabilized, the estimated and experimental values will probably be very close, like those observed for 5 Hz and 6 Hz. This result means that by measuring the mechanical part’s temperature rise, it is possible to estimate both its stiffness increase (force to produce the same deflection) and the loading frequency.

## 4. Conclusions

The temperature dependence of the dynamic superelastic response of a NiTi Belleville spring was analyzed. Thermomechanical coupling in cyclic compression tests with a fixed deflection amplitude at various loading frequencies was monitored by a thermocouple installed on the surface of the spring. The critical frequency of self-heating (*f_c_*) was determined by a proposed methodology using superelastic cycling at different frequencies. The temperature variation during cycling was analyzed, confirming the internal temperature increase of the NiTi Belleville spring. The proposed methodology consists in identifying the frequency at which there is no difference in the force at the end of loading between the first and last superelastic cycles (128 cycles were used). Keeping the same environmental conditions, cycling above this frequency will cause the self-heating of the SMA device. For the dimensions and NiTi alloy used in the Belleville spring of this study, this frequency was of the order of 1.7 Hz.

The latent heat accumulated during the forward and reverse phase transformations increases the internal temperature of the SMA, resulting in stiffer superelastic loops. Consequently, peak forces increase, and functional properties, such as dissipated energy per cycle and the equivalent viscous damping factor decrease. This behavior is attributed to the Clausius–Clapeyron law which correlates the stress for phase transformation with temperature through a linear relationship. Due to this relation, the self-heating in SMA devices working under dynamic conditions is often unavoidable, under weak heat dissipation conditions. Therefore, the effects of self-heating on the functional properties must be considered in the design of SMA dynamic devices. This was demonstrated based on a theoretical estimate of the increase in force with increasing temperature of the Belleville spring, from a numerical simulation to determine the Clausius-Clayperon coefficient. This estimate worked very well for the frequencies of 5 Hz and 6 Hz when the temperature increase reached a permanent regime.

## Figures and Tables

**Table 1:** Mechanical properties used in FEA.

Body (Material Model)	Material Parameter	Value		
Compression plates (Linear elastic)	E (MPa)	200,000		
	ν	0.3		
NiTi Belleville spring (SMA superelastic)	E (MPa)	40,000		
	ν	0.3		
		35 °C	45 °C	55 °C
	$\sigma_{s}^{\mathrm{AM}}$ (MPa)	350	450	525
	$\sigma_{f}^{\mathrm{AM}}$ (MPa)	575	625	650
	$\sigma_{s}^{\mathrm{MA}}$ (MPa)	300	350	375
	$\sigma_{f}^{\mathrm{MA}}$ (MPa)	150	175	200
	$\epsilon_{L}$ (mm/mm)	0.06		
	α	0		

## Data Availability

The data presented in this study are available on request from the corresponding author.
